# Integrative molecular subtyping and diagnostic model construction for Moyamoya disease based on diverse programmed cell death gene patterns

**DOI:** 10.1186/s13023-025-03816-y

**Published:** 2025-06-03

**Authors:** Qikai Tang, Chenfeng Ma, Hu Li, Jiaheng Xie, Weiqi Bian, Qingyu Lu, Zeyu Wan, Wei Wu

**Affiliations:** 1 Department of Neurosurgery, Jiangsu Province Hospital, The First Affiliated Hospital of Nanjing Medical University, Nanjing, Jiangsu 210029 China; 2 Department of Neurosurgery, People’s Hospital of Ganyu District, Lianyungang, China; 3 Department of Plastic Surgery, Xiangya Hospital, Central South University, Changsha, 410008 P. R. China; 4 Department of Neurosurgery, Affiliated Hospital of Yifu, Nanjing Medical University, Nanjing, Jiangsu P. R. China

**Keywords:** Moyamoya disease, Bioinformatics, WGCNA, RNA-sequencing, Biomarker

## Abstract

**Background:**

The role of cell death in the pathogenesis of Moyamoya disease (MMD) remains unclear.

**Methods:**

Gene expression data from two publicly available MMD datasets (GSE157628 and GSE189993) were integrated. Differential expression analysis of cell death-related genes was performed, followed by immune cell infiltration analysis using the CIBERSORT algorithm. Unsupervised clustering identified molecular subgroups within MMD patients, and these were further analyzed using Gene Set Variation Analysis (GSVA) and Weighted Gene Co-expression Network Analysis (WGCNA). Diagnostic models were constructed using machine learning algorithms, and key model genes were validated by qRT-PCR in arterial samples from MMD patients.

**Results:**

Significant differences in gene expression were observed, with immune cell infiltration analysis showing differences in T follicular helper cells, activated dendritic cells, resting and activated mast cells, and eosinophils. Unsupervised clustering identified two distinct patient groups, and differential gene expression revealed upregulation of genes like ADRB2, FGR, ICAM1, IL1B, DDIT4, CXCL1, CASP4, G0S2, CYP1B1, and CD74 in C2 cluster. Weighted gene co-expression network analysis (WGCNA) identified key genes in the most correlated module. Machine learning models (RF, SVM, GLM, XGB) were constructed, with the SVM model showing the best performance (AUC = 1.000). A nomogram based on the SVM model demonstrated high predictive accuracy. Enrichment analysis revealed that key genes were involved in apoptosis and antigen processing, with strong diagnostic performance confirmed by ROC curves. PCR analysis revealed that model genes RGS1, MUC1, KCNA2, TAC1, and SOST were all up-regulated in MMD group.

**Conclusions:**

This study provides novel insights into the molecular landscape of MMD, highlighting the importance of cell death-related pathways and immune responses. The identified biomarkers and molecular subgroups offer potential targets for therapeutic intervention and improved diagnostic strategies in MMD.

**Supplementary Information:**

The online version contains supplementary material available at 10.1186/s13023-025-03816-y.

## Introduction

Moyamoya disease (MMD) is a progressive cerebrovascular disorder characterized by the stenosis or occlusion of the internal carotid arteries and the subsequent formation of abnormal collateral vessels at the base of the brain [1–3]. The disease is most prevalent in East Asian populations, particularly among children and young adults [4]. However, its etiology remains poorly understood, with both genetic and environmental factors implicated in its pathogenesis [5]. MMD is associated with a high risk of ischemic stroke, intracranial hemorrhage, and cognitive decline, making it a significant clinical challenge [6]. Current treatment strategies, such as revascularization surgery, aim to prevent stroke, but the molecular mechanisms underlying MMD have yet to be fully elucidated [7].

Recent studies have highlighted the role of cellular death processes, including apoptosis, necroptosis, and ferroptosis, in cerebrovascular diseases [8–10]. These processes are intricately linked to the inflammatory response and immune cell infiltration, which play crucial roles in the progression of MMD [11]. However, the specific contributions of cell death-related genes and their interaction with immune cells in MMD remain largely unexplored [12]. Given the potential significance of these genes in the pathogenesis of MMD, a comprehensive analysis of their expression patterns and related immune infiltration could provide valuable insights into the disease’s molecular mechanisms and suggest new therapeutic targets.

The aim of this study was to identify potential molecular subgroups and therapeutic targets related to cell death pathways and immune responses in MMD. Our findings provide novel insights into the molecular landscape of MMD and lay the groundwork for future therapeutic interventions.

## Methods

### High-throughput sequencing data download

In this study, the datasets GSE157628 and GSE189993 were downloaded from the Gene Expression Omnibus (GEO) database [13]. MMD was defined based on clinical diagnosis supported by imaging findings of bilateral stenosis or occlusion of the internal carotid arteries (ICA) and its major branches, with the formation of collateral vessels, as observed in cerebral angiography. Patients with other possible diagnoses, including cerebral vascular malformations, intracranial aneurysms, or other conditions leading to similar symptoms, were excluded from this analysis. Middle cerebral artery tissue samples from patients with moyamoya disease constituted the disease group of the dataset, while those obtained surgically for internal carotid aneurysms/epilepsy constituted the control group. The data files, including raw expression matrices and sample annotation information, were downloaded using the standardized data retrieval interface provided by GEO. Specifically, the GEO website was accessed, and the datasets were retrieved using their unique identifiers (GSE157628 and GSE189993). To ensure data integrity and accuracy, the raw data underwent standardized processing by the GEO database, including background correction and normalization. All genes associated with cell death were summarized from previously published literature, as listed in Supplementary Table 1.

### Differential expression analysis

Initially, a list of genes involved in 18 types of cell death mechanisms was extracted from Supplementary Table 1. These genes represent various cell death pathways, including but not limited to apoptosis, autophagy, pyroptosis, necroptosis, and ferroptosis. This gene list served as the genes of interest for subsequent differential expression analysis and visualization. Gene expression data from the GSE157628 and GSE189993 datasets were merged. To ensure data consistency, both datasets underwent background correction and normalization prior to merging. The ComBat algorithm was applied to correct batch effects, eliminating systematic biases caused by differences in experimental conditions. The merged dataset included gene expression values from all samples, providing a comprehensive data foundation for further analysis. A linear model was used to perform differential expression analysis on the genes within the merged dataset. For each gene, the log2 fold change (log2FC) and p-value were calculated based on different experimental groups or conditions (e.g., disease state vs. control). The Benjamini-Hochberg method was employed to adjust for multiple testing, yielding adjusted p-values (adj.p-values) to identify significantly differentially expressed genes. The selection criteria for differentially expressed genes were|log2FC| > 1 and adj.p-value < 0.05. Finally, the ComplexHeatmap package was used to visualize these differentially expressed genes through a complex heatmap.

### Correlation and chromosomal analysis of cell death genes

Correlation analysis and visualization were performed using the “corrplot” package in R to illustrate the association patterns of gene expression. Additionally, the chromosomal locations of cell death genes (CDGs) were obtained using Perl scripts and the “RCircos” package in R. These tools were used to construct a genomic map showing the distribution of CDGs across different chromosomes. These analyses help to understand the mechanisms by which CDGs interact with immune cells and provide a reference for their genomic location.

### Immune infiltration analysis

To further explore differences in immune infiltration between Moyamoya disease and control groups, the CIBERSORT algorithm was applied to quantify the composition of immune cells in the samples. CIBERSORT is a computational method based on linear support vector regression, used to estimate the proportions of specific immune cell types in complex tissues. First, gene expression profiles were extracted from the merged GSE157628 and GSE189993 datasets. To ensure the accuracy of the analysis, the standard LM22 gene set provided by the CIBERSORT website was selected, which includes signature genes for 22 common immune cell types. The expression matrix of the samples was matched with the LM22 gene set, and the data were standardized before being input into the CIBERSORT tool to analyze the immune cell composition in the samples. CIBERSORT calculated the relative proportions of each immune cell type in the samples by regressing the expression data against the LM22 gene set. Default parameters were used in the mixture model, and 1000 permutations were run to enhance the robustness and reliability of the results. The analysis results included the relative percentages of 22 immune cell types in each sample, as well as the p-values for each cell type, used to assess their significance across different groups.

### Unsupervised consensus clustering analysis

To investigate the expression patterns of cell death genes in Moyamoya disease and their relationship with sample classification, unsupervised consensus clustering was applied to classify the samples. This analysis was based on the expression data of the cell death genes identified earlier. First, the gene expression data of all samples were standardized to ensure data consistency and comparability. The z-score normalization method was used to convert each gene’s expression values into a standard normal distribution, eliminating differences in scale across gene expression levels. The ConsensusClusterPlus package in R was used for unsupervised consensus clustering. Different cluster numbers (k = 2 to k = 9) were tested, with the consensus matrix calculated for each k value to evaluate sample stability across different cluster numbers. The cumulative distribution function (CDF) curve and its relative change (Delta area) were used to determine the optimal number of clusters. Based on the results, k = 2 was selected as the optimal number of clusters, dividing the samples into two main subgroups. After identifying the C1 and C2 subgroups, edgeR and DESeq2 packages in R were used to perform differential gene analysis between the two subgroups. The gene expression data were first standardized, and then differentially expressed genes were identified based on the log2 fold change (log2FC) and p-value. The Benjamini-Hochberg method was applied for multiple testing correction, with|log2FC| > 1 and adj.p-value < 0.05 used as the criteria for selecting significantly differentially expressed genes.

### GSVA enrichment analysis

To explore the potential biological pathways and functional differences between the two subgroups, Gene Set Variation Analysis (GSVA) was used for functional enrichment analysis. GSVA is an unsupervised method that assesses the activation state of gene sets by calculating enrichment scores across predefined gene sets for each sample. Based on the gene sets from the Molecular Signatures Database (MSigDB), enrichment scores were calculated for each sample and the differences between the C1 and C2 subgroups were compared. Significant gene sets were visualized using a heatmap to reveal potential functional differences between the two groups.

### Weighted gene co-expression network analysis (WGCNA)

To further explore the co-expression patterns of cell death genes and their functional associations in the C1 and C2 subgroups, Weighted Gene Co-expression Network Analysis (WGCNA) was performed. First, a similarity matrix of the gene expression matrix was constructed, which was then converted into a weighted adjacency matrix using the soft-threshold method. Next, dynamic tree cutting was applied to cluster genes into modules, and the correlation between modules and sample traits was calculated. Modules highly correlated with subgroup-specific traits were prioritized, and enrichment analysis of genes within these modules was conducted to identify key genes and their associated biological processes. The WGCNA analysis identified gene modules and hub genes potentially playing critical roles in the pathogenesis of Moyamoya disease.

### Construction of diagnostic models using machine learning algorithms

After identifying key module genes through WGCNA analysis, various machine learning methods were used to construct diagnostic models for Moyamoya disease. Random Forest (RF) is an ensemble learning method that constructs multiple independent decision trees for classification or regression. The Support Vector Machine (SVM) model generates a hyperplane in the feature space to effectively separate data points. The Generalized Linear Model (GLM) extends multiple linear regression to provide a more efficient and flexible approach for analyzing the relationship between the dependent variable and categorical or continuous independent variables. eXtreme Gradient Boosting (XGB) is a gradient boosting-based ensemble tree algorithm used to balance classification error and model complexity. All machine learning models were executed with default parameters and evaluated comprehensively using 5-fold cross-validation. The “DALEX” package in R was used to interpret the four machine learning models, visualizing their residual distribution and feature importance. Additionally, the “pROC” package was used to plot the Area Under the Curve (AUC) of the Receiver Operating Characteristic (ROC) curve. The best-performing machine learning model was selected based on the model’s performance, with the top five genes in the model used as predictive indicators for diagnosing Moyamoya disease.

### Nomogram construction

To further quantify and visualize the predictive ability of the diagnostic model for Moyamoya disease, a nomogram was constructed based on the output of the best-performing machine learning model. A nomogram is a visualization tool for individualized prediction that quantifies the effects of multiple variables into scores and maps them to a total score to calculate the probability of an event. Using the “rms” package in R, a nomogram was constructed based on the important feature genes in the best-performing model. By summing the scores of each gene in the nomogram, the risk of Moyamoya disease occurrence can be predicted, providing a basis for clinical decision-making.

### ROC curve and AUC value calculation for model genes

To evaluate the predictive performance of each gene in the model, the ROC curve and the AUC values were calculated for the five important model genes. The ROC curve reflects the sensitivity and specificity of a classification model at different thresholds, while the AUC value quantifies the overall performance of the model. The “pROC” package in R was used to plot the ROC curve for each gene and calculate the AUC value. The AUC values range from 0.5 to 1, with values closer to 1 indicating higher predictive accuracy for the disease. By comparing the AUC values of the five model genes, the gene with the best predictive performance for diagnosing Moyamoya disease was identified.

### PCR validation of model genes

Arteriae temporalis superficialis(ATS) samples were obtained from moyamoya disease patients (experimental group). Blood vessel samples from necrotic tissue from traumatic brain injury (TBI) patients were normal controls. Ethical approval and informed consent were secured prior to sample collection. Total RNA was extracted from the tissues and reverse transcribed into cDNA. qRT-PCR was performed to assess the expression levels of model genes. ACTB served as the internal control for normalization. The primer sequence used is in Supplemental Table 2.

## Results

### Differential gene expression and immune cell infiltration analysis

**Fig. 1:**
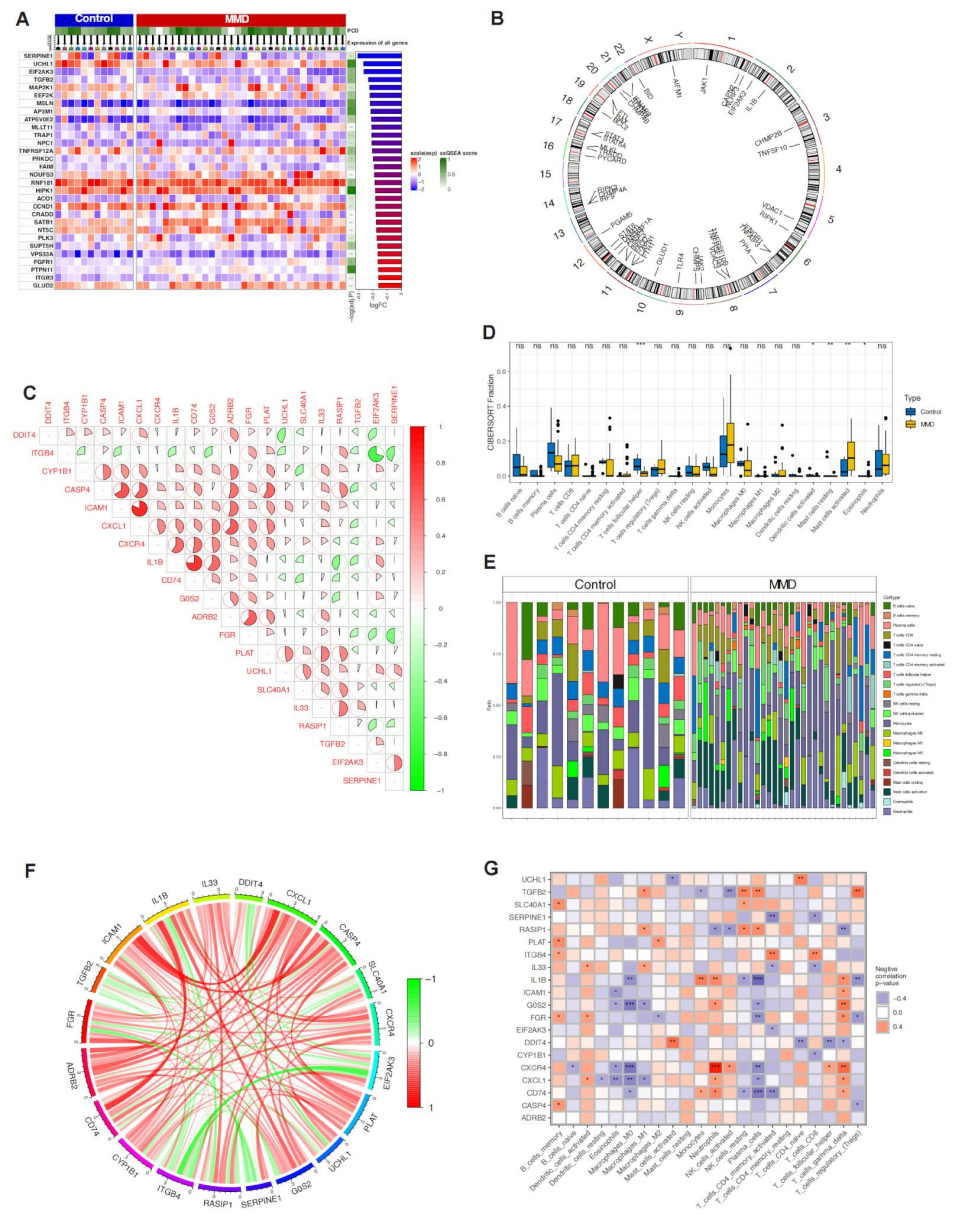
Differential Gene Expression and Immune Cell Infiltration in Moyamoya Disease (MMD). (**A**) Heatmap showing the differential expression of cell death-related genes between the MMD group and the control group after merging the GSE157628 and GSE189993 datasets. (**B**) Chromosomal localization of differentially expressed genes. (**C**) Correlation matrix depicting the relationships among differentially expressed genes. (**D**, **E**) Immune cell infiltration analysis showing significant differences in the infiltration abundance of T cells follicular helper, Dendritic cells activated, Mast cells resting, Mast cells activated, and Eosinophils between the MMD and control groups. (**F**) Additional correlation matrix illustrating the interrelationships of differentially expressed genes. (**G**) Heatmap displaying the correlation between differentially expressed genes and various immune cell types

First, after merging the GSE157628 and GSE189993 datasets, we analyzed the differential expression of cell death-related genes between the MMD group and the control group, displaying the results in a heatmap. The results showed that multiple genes were differentially expressed between the MMD and control groups (Fig. 1A). Additionally, we presented the chromosomal localization of these genes (Fig. 1B). Figure 1C and F show the correlations between differentially expressed genes. Furthermore, we performed an immune cell infiltration analysis, and the results indicated significant differences in the infiltration abundance of T cells follicular helper, Dendritic cells activated, Mast cells resting, Mast cells activated, and Eosinophils between the two groups (Fig. 1D and E). The immune-related heatmap also showed significant correlations between these genes and various immune cells (Fig. 1G). These analyses preliminarily suggest the potential significance of cell death in MMD.

### Unsupervised clustering analysis

**Fig. 2:**
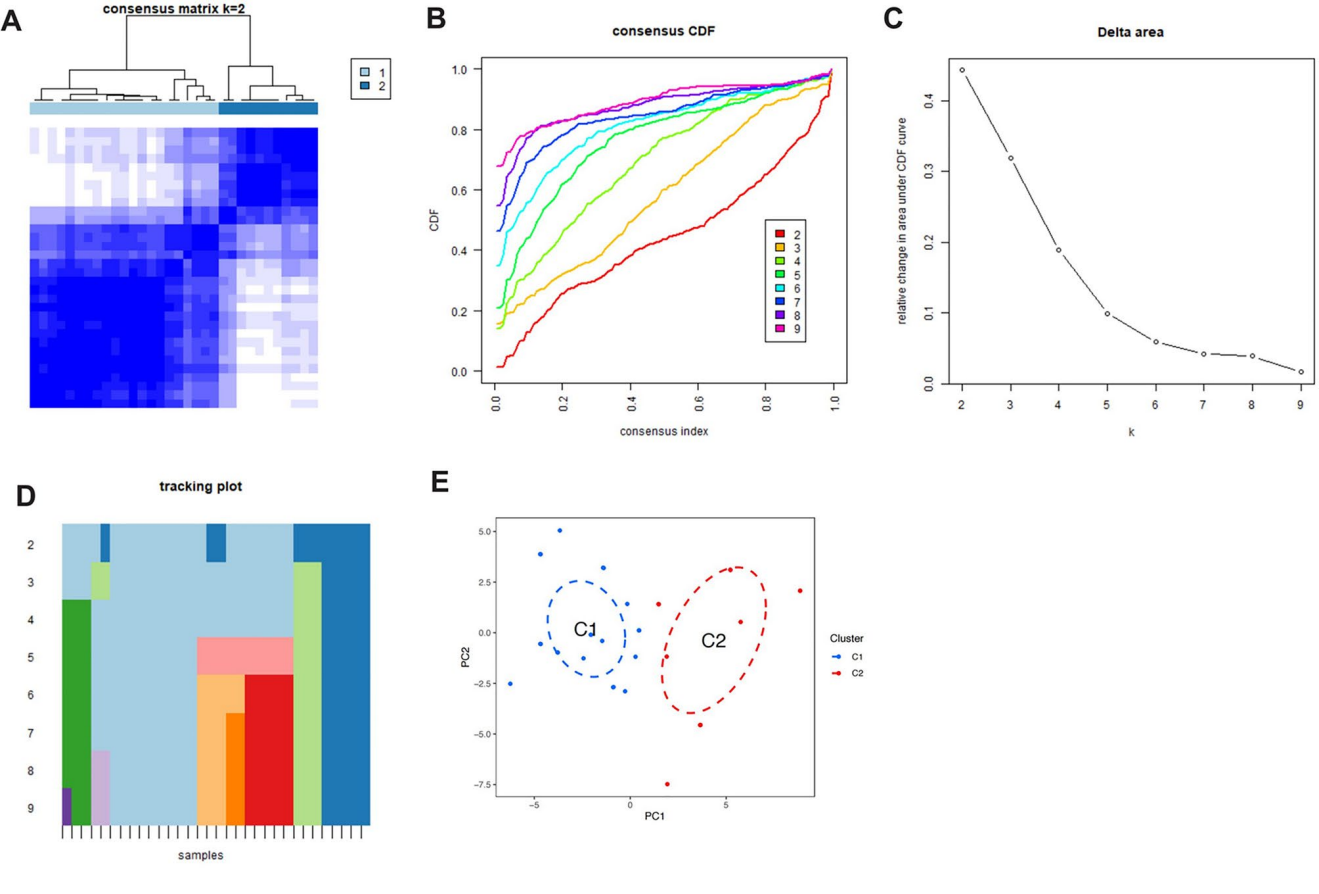
Unsupervised Clustering Analysis of MMD Patients Based on Cell Death-Related Genes. (**A**) Consensus clustering heatmap for k = 2, indicating the optimal number of clusters. (**B**, **C**, **D**) CDF curve and delta area plot demonstrating stable clustering at k = 2. (**E**) Principal component analysis (PCA) showing clear separation of MMD patients into two distinct clusters

Based on cell death-related genes, we determined that the optimal number of clusters for MMD patients was k = 2, and the patients were clustered into two groups (Fig. 2A). The CDF curve also showed stable clustering when k = 2 (Fig. 2B, C and D). Further principal component analysis revealed that under these clustering conditions, patients were well separated into two categories (Fig. 2E).

### Differential gene expression and immune infiltration analysis in cell death clusters

**Fig. 3:**
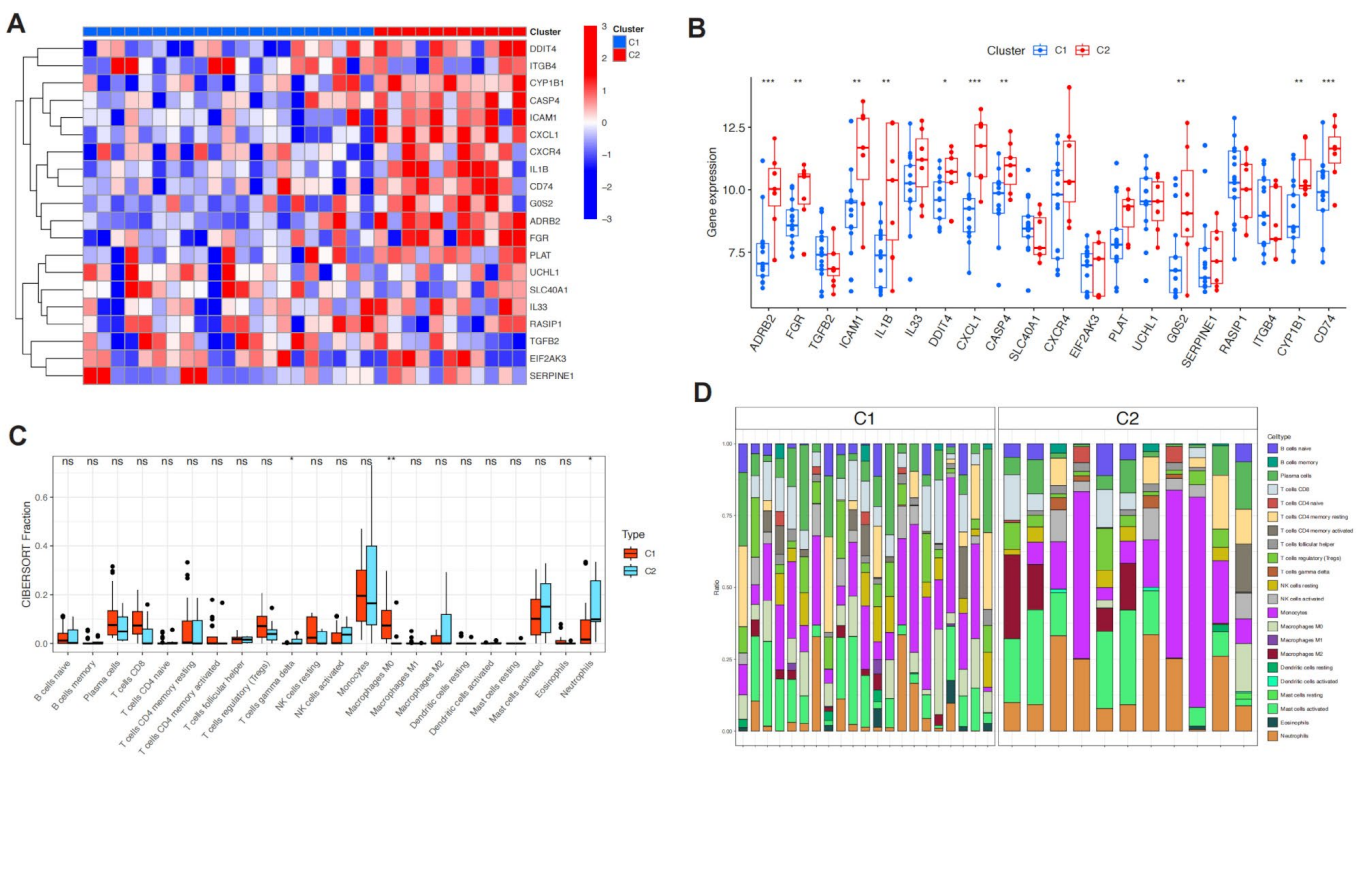
Differential Gene Expression and Immune Infiltration Analysis Between Cell Death Clusters. (**A**) Heatmap showing differential gene expression between the two cell death clusters, C1 and C2. (**B**) Bar plot highlighting significantly upregulated genes in C2, including ADRB2, FGR, ICAM1, IL1B, DDIT4, CXCL1, CASP4, G0S2, CYP1B1, and CD74. (**C**, **D**) Immune cell infiltration analysis between C1 and C2, showing differences in the abundance of T cells gamma delta, Macrophages M0, and Neutrophils

**Fig. 4:**
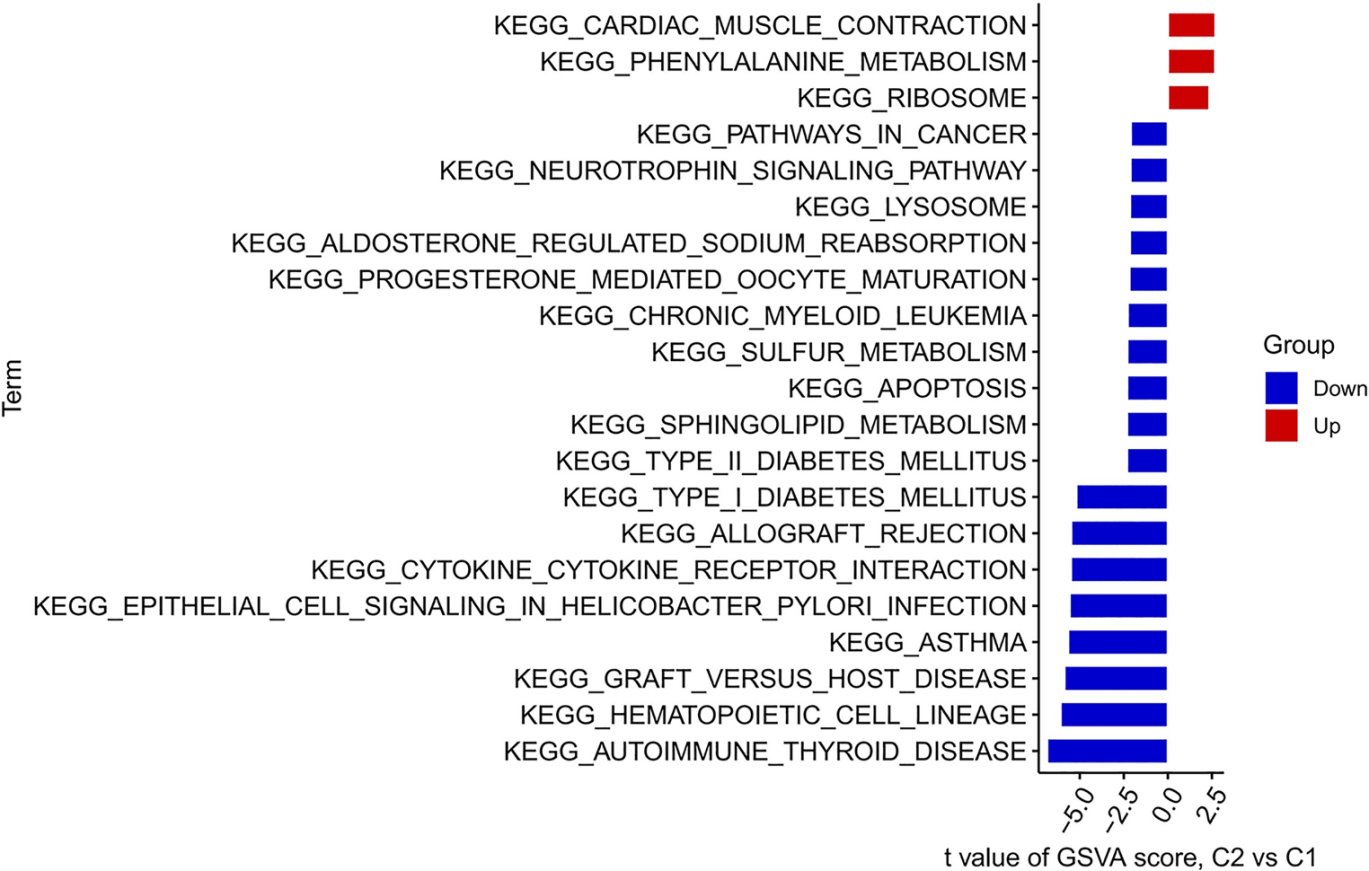
GSVA Enrichment Analysis of Cellular Pathways Between Cell Death Clusters. GSVA enrichment analysis highlighting significant differences in cellular pathways between C1 and C2, including Phenylalanine metabolism

After obtaining the cell death clusters, we analyzed the differential gene expression between C1 and C2 and presented the results in a heatmap (Fig. 3A). We found that ADRB2, FGR, ICAM1, IL1B, DDIT4, CXCL1, CASP4, G0S2, CYP1B1, and CD74 were significantly upregulated in C2 (Fig. 3B). Additionally, there were significant differences in immune cell infiltration between C1 and C2, including T cells gamma delta, Macrophages M0, and Neutrophils (Fig. 3C and D). GSVA enrichment analysis revealed significant differences in cellular pathways between C1 and C2, including Phenylalanine metabolism (Fig. 4).

### Weighted gene co-expression network analysis (WGCNA)

**Fig. 5:**
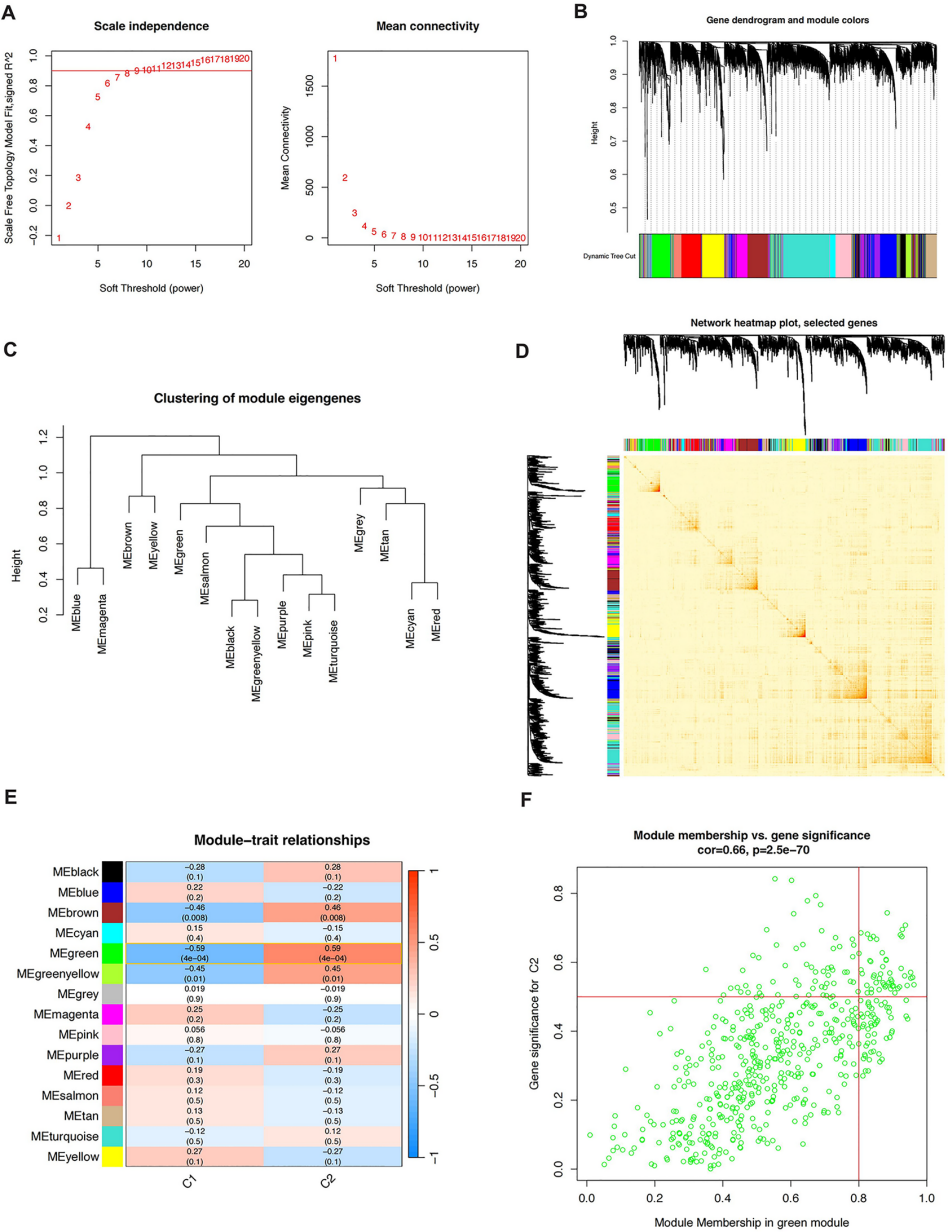
Weighted Gene Co-expression Network Analysis (WGCNA) Based on the Cell Death Clustering Model. (**A**) Determination of the optimal soft threshold power for WGCNA, showing an R² value of 0.9 at a soft threshold of 9. (**B**) Clustering dendrogram of MMD patients. (**C**) Heatmap of module-trait relationships showing the correlation between gene modules and cell death clusters. (**D**) Cluster dendrogram of genes with dissimilarity based on topological overlap, along with the corresponding module colors. (**E**, **F**) Correlation between the green module and the cell death clustering model, with key genes extracted for further analysis

Based on the unsupervised clustering model of cell death, we performed a weighted gene co-expression network analysis. First, we identified the optimal soft threshold as 9, with an R^2 of 0.9 (Fig. 5A), where the sample clustering effect was best, and the genes were divided into different colored modules based on expression patterns (Fig. 5B and D). Among them, the green module was found to have the strongest correlation with the clustering model, and the genes within this module were extracted as key genes in WGCNA (Fig. 5E and F).

### Machine learning for diagnostic model construction

**Fig. 6:**
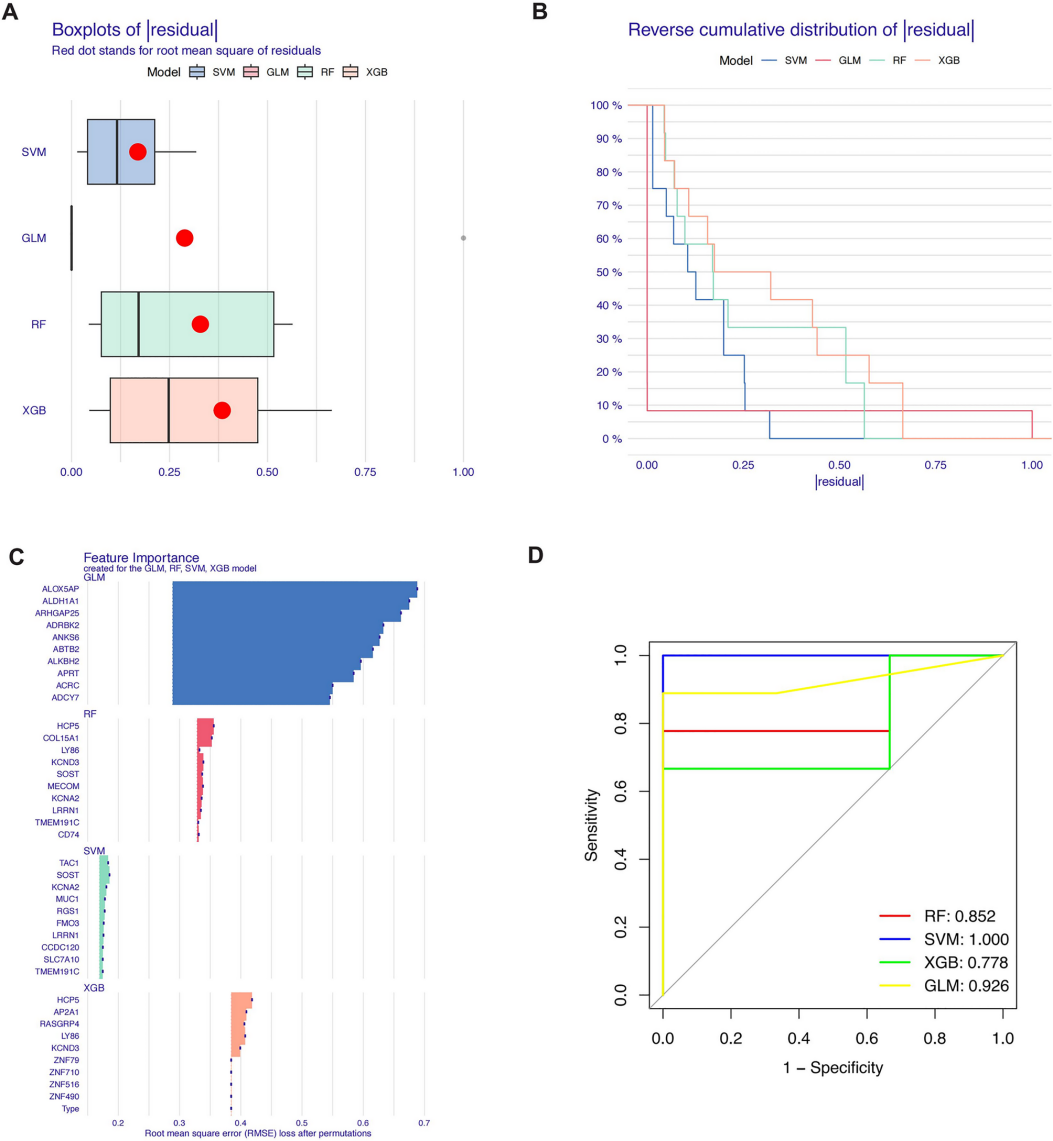
Machine Learning for Diagnostic Model Construction Using Green Module Genes. (**A**, **B**) Residual analysis of four machine learning models (RF, SVM, GLM, XGB) constructed using green module genes and 449 genes from GSE157628 and GSE189993, with SVM showing the lowest residuals. (**C**) Ranking of the top 10 significant genes for each model based on RMSE. (**D**) ROC curves for the four models, with the SVM model achieving the highest AUC (1.000), indicating superior discriminatory performance

To further identify specific genes with high diagnostic value, we used four machine learning algorithms—RF, SVM, GLM, and XGB—to construct diagnostic models using the green module genes and the 449 genes from GSE157628 and GSE189993. The “DALEX” package was used to compare these four models and analyze the residual distribution of each. The results indicated that the SVM model had the lowest residuals (Figs. 6A, B). The top 10 significant genes for each model were ranked based on RMSE (Fig. 6C). We then plotted ROC curves for the four models based on 5-fold cross-validation to comprehensively evaluate their discriminatory performance. The AUCs for the four models were: RF: AUC = 0.852; SVM: AUC = 1.000; XGB: AUC = 0.778; and GLM: AUC = 0.926 (Fig. 6D). In conclusion, the SVM model better distinguished different patient groups. The five significant genes in the SVM model—TAC1, SOST, KCNA2, MUC1, and RGS1—were identified as predictive genes for subsequent analysis.

### Nomogram construction and validation

**Fig. 7:**
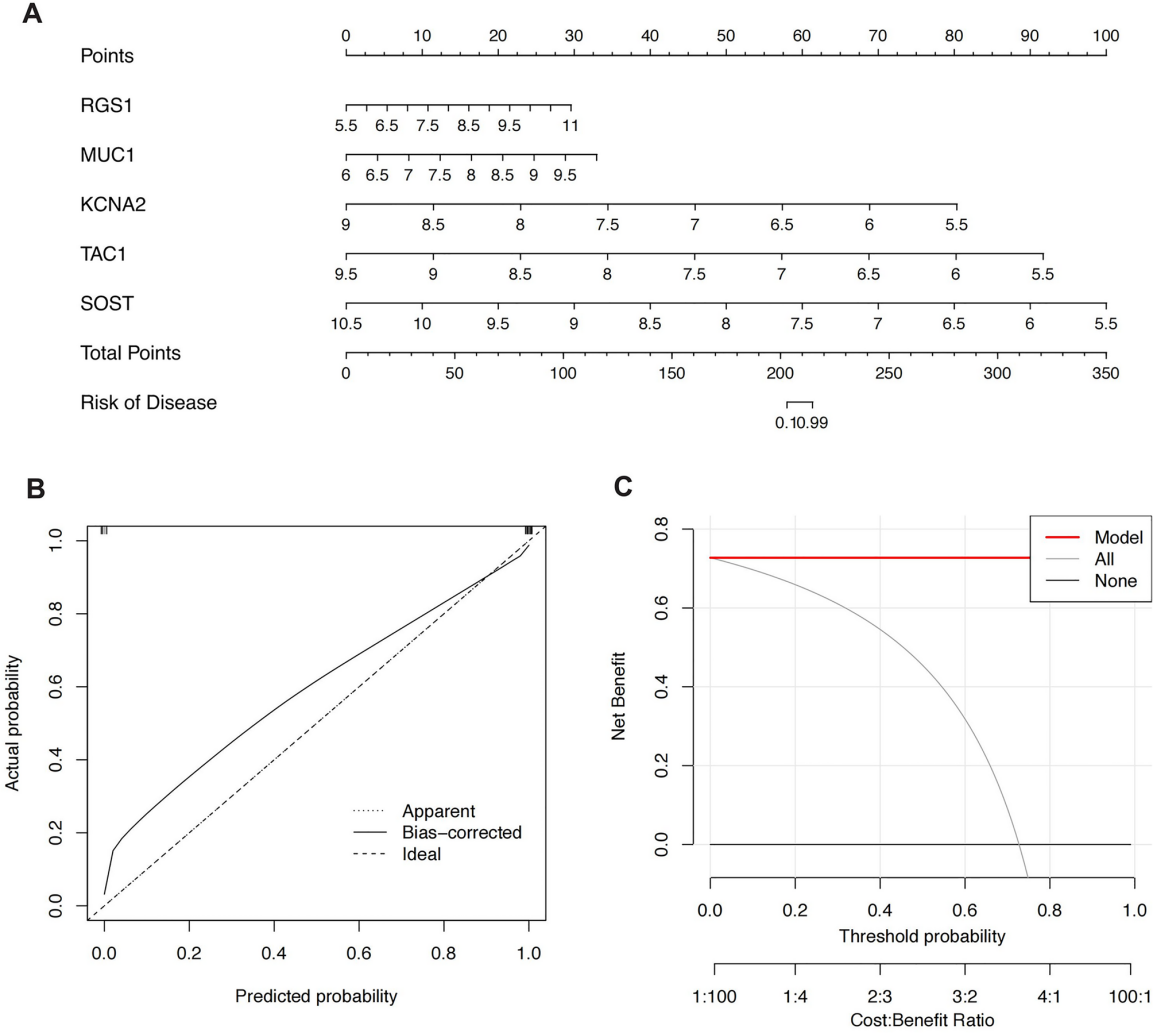
Nomogram Construction and Validation Based on the SVM Model. (**A**) Nomogram constructed for predicting MMD patient outcomes based on the SVM model. (**B**) Calibration curve showing a minimal margin of error between actual and predicted risks. (**C**) Decision curve analysis (DCA) indicating high accuracy of the nomogram for clinical decision-making

We constructed a nomogram to further evaluate the predictive efficacy of the SVM model (Fig. 7A). The calibration curve and DCA were used to evaluate the predictive efficiency of the constructed nomogram model. The calibration curve showed a small margin of error between the actual risk and predicted risk for RA clustering (Fig. 7B), and the DCA results indicated that the nomogram had high accuracy, providing a reference for clinical decision-making (Fig. 7C).

### Enrichment analysis and diagnostic performance of key genes in the model

**Fig. 8:**
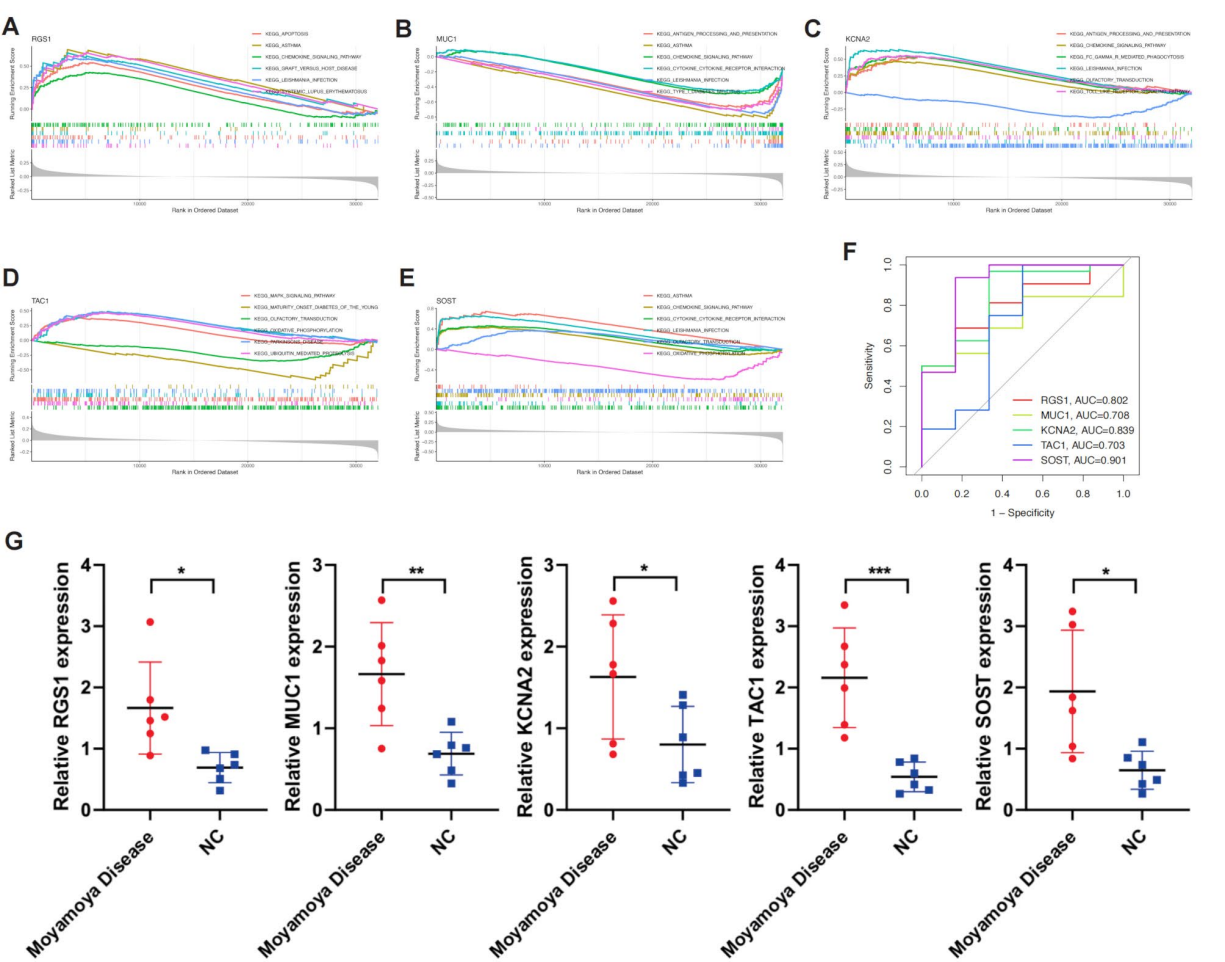
Enrichment Analysis and Diagnostic Performance of Key Genes in the SVM Model. (**A**-**E**) GSEA enrichment analysis for the key genes RGS1, MUC1, KCNA2, TAC1, and SOST, highlighting their association with critical pathways such as apoptosis and antigen processing and presentation. (**F**) ROC curves for each key gene, demonstrating high AUC values and strong diagnostic performance. (**G**) PCR validation of RGS1, MUC1, KCNA2, TAC1, and SOST expression, showing upregulation in MMD samples compared to controls

To further understand the significance of the key genes in the SVM model, we performed GSEA enrichment analysis to explore the functions and pathways involved. As shown in Fig. 8A and E, the key genes RGS1, MUC1, KCNA2, TAC1, and SOST were found to be significantly associated with several key pathways, including apoptosis and antigen processing and presentation pathways. Additionally, we constructed ROC curves for these five key genes separately and calculated the area under the curve, revealing that all five genes had high AUC values, indicating their strong diagnostic performance (Fig. 8F). Finally, we verified the expression of key model genes using PCR. RGS1, MUC1, KCNA2, TAC1, and SOST were all found to be up-regulated in moyamoya disease (Fig. 8G).

## Discussion

Our study provides a comprehensive analysis of cell death-related genes and immune cell infiltration in MMD, revealing key molecular mechanisms that may contribute to the pathogenesis of this complex disorder [14]. The differential expression analysis identified several cell death-related genes that were significantly altered between MMD patients and healthy controls, underscoring the potential involvement of apoptotic, necroptotic, and ferroptotic pathways in MMD [15]. Notably, the immune cell infiltration analysis revealed significant differences in the abundance of several immune cell types, including T cells follicular helper, activated dendritic cells, and mast cells, between the MMD and control groups. These findings suggest that cell death-related processes may drive the inflammatory response and immune dysregulation observed in MMD, potentially contributing to disease progression [16, 17].

The unsupervised clustering analysis based on cell death-related genes further identified two distinct molecular subgroups of MMD patients, each with unique gene expression profiles and immune cell infiltration patterns. The clustering analysis was supported by principal component analysis, which demonstrated clear separation between the two patient subgroups. The significant upregulation of genes such as ADRB2, FGR, ICAM1, and IL1B in one of the subgroups (C2) suggests that these patients may exhibit a more pronounced inflammatory phenotype, which could be linked to a higher risk of ischemic events. The differences in immune cell infiltration between the two subgroups, particularly in T cells gamma delta, M0 macrophages, and neutrophils, further highlight the heterogeneity of immune responses in MMD, which may have implications for patient stratification and personalized treatment approaches [18–20].

Importantly, GSVA enrichment analysis between the two molecular clusters (C1 and C2) revealed significant differences in several biological pathways, including phenylalanine metabolism. Phenylalanine, as an essential aromatic amino acid, is involved in various physiological processes including neurotransmitter synthesis, oxidative stress regulation, and vascular function [21]. Abnormal phenylalanine metabolism has been linked to endothelial dysfunction and increased oxidative stress, both of which are implicated in vascular remodeling—a central pathological feature of Moyamoya disease [22, 23]. In particular, the accumulation of phenylalanine or its metabolites can induce metabolic stress in vascular endothelial cells, potentially triggering inflammatory signaling and apoptotic pathways. These mechanisms may synergize with the observed immune dysregulation and cell death processes, thereby exacerbating vascular stenosis and collateral vessel formation in MMD. Thus, the GSVA findings suggest that dysregulated phenylalanine metabolism might contribute to MMD pathogenesis through metabolic and vascular remodeling-related mechanisms, providing a novel angle for future mechanistic exploration.

Our WGCNA identified key gene modules associated with the cell death-related clustering model, with the green module showing the strongest correlation. The identification of hub genes within this module provides potential targets for further investigation, as these genes may play pivotal roles in the molecular networks driving MMD pathogenesis. Furthermore, the use of machine learning algorithms to construct diagnostic models based on these key genes demonstrated the superior performance of the SVM model, which achieved the highest accuracy and AUC. The identification of five significant genes—TAC1, SOST, KCNA2, MUC1, and RGS1—as predictive markers highlights their potential utility in improving diagnostic precision and patient stratification in MMD [24]. These identified genes—TAC1, SOST, KCNA2, MUC1, and RGS1—have well-established roles in vascular diseases, primarily through their involvement in inflammation, immune regulation, vascular remodeling, and neuroprotection. In the context of MMD, these genes may contribute to the disease through mechanisms such as modulation of blood vessel plasticity, inflammatory responses, and ischemic injury. Specifically, TAC1 may influence vascular remodeling, SOST may regulate vascular calcification and smooth muscle cell proliferation, KCNA2 could impact neuronal excitability and vascular function, MUC1 might affect endothelial cell function and immune regulation, and RGS1 could modulate immune cell activation and migration [25–29]. These insights suggest that these genes may play pivotal roles in MMD pathogenesis, offering potential targets for future therapeutic strategies. Further research is needed to validate these findings and elucidate their specific contributions to MMD development.

In the context of known biomarkers for MMD, such as RNF213 mutations, circulating endothelial progenitor cells (EPCs), and inflammatory cytokines including VEGF and TGF-β, the newly identified genes—TAC1, SOST, KCNA2, MUC1, and RGS1—represent a novel set of molecular indicators with both diagnostic and mechanistic potential [30]. While traditional markers like RNF213 are primarily associated with genetic susceptibility and have limited diagnostic sensitivity outside East Asian populations, our study highlights a complementary and potentially more universally applicable panel based on transcriptomic alterations and immune-metabolic dysregulation. Moreover, the SVM-based model built on these five genes demonstrated superior diagnostic performance (AUC > 0.95) compared to conventional biomarkers reported in previous studies, suggesting a greater potential for clinical implementation.

Importantly, these new markers not only reflect systemic immune and metabolic changes that characterize MMD but also capture heterogeneity within the patient population, as evidenced by their distinct expression patterns across molecular subclusters. This capacity for stratification may help guide future risk prediction and individualized therapeutic strategies. Therefore, integrating these novel biomarkers with existing markers could enhance diagnostic precision and facilitate a more nuanced understanding of MMD pathogenesis. Future prospective studies are warranted to validate the additive or synergistic value of these markers in clinical cohorts.

Finally, the construction of a nomogram based on the SVM model provided a practical tool for assessing the diagnostic value of these key genes. The calibration curve and DCA confirmed the model’s accuracy and clinical utility, suggesting that this approach could be integrated into routine clinical practice to guide treatment decisions. Moreover, the GSEA enrichment analysis revealed that the identified key genes are involved in critical pathways such as apoptosis and antigen processing, further supporting their relevance in MMD pathogenesis. The robust diagnostic performance of these genes, as evidenced by their high AUC values, underscores their potential as biomarkers for early detection and monitoring of disease progression.

In conclusion, our study sheds light on the intricate interplay between cell death-related genes and immune responses in MMD, providing new insights into the molecular underpinnings of this disorder. The identification of distinct molecular subgroups within MMD patients and the development of a highly accurate diagnostic model based on key genes highlight the potential for precision medicine approaches in this field. Future research should focus on validating these findings in larger cohorts and exploring the therapeutic implications of targeting cell death pathways and immune responses in MMD.

Despite the strengths of this study, several limitations should be acknowledged. First, the sample size is relatively small, which may affect the generalizability of our findings. Although the results were consistent across merged GEO datasets, validation in larger, independent cohorts is essential to confirm the robustness and clinical applicability of the identified biomarkers and molecular subtypes. Second, the study is primarily based on bioinformatics analyses; while these approaches provide valuable insights into potential mechanisms, functional validation through in vitro and in vivo experiments is necessary to elucidate the exact biological roles of the identified genes, particularly in the context of vascular remodeling, immune regulation, and cell death in Moyamoya disease. Third, due to limitations in available clinical metadata, we were unable to correlate gene expression patterns with detailed clinical parameters such as Suzuki stages or treatment outcomes, which would further enhance the translational relevance of our findings. Addressing these constraints in future studies will be critical for advancing these biomarkers toward clinical application.

## Electronic supplementary material

Below is the link to the electronic supplementary material.

Supplementary Material 1

Supplementary Material 2

## Data Availability

The datasets analyzed in this study were downloaded and accessed from the Gene Expression Omnibus (GEO) database: https://www.ncbi.nlm.nih.gov/geo/, with accession No: GSE157628 and GSE189993.
